# Supplementary material for: Shape-recovery of implanted shape-memory devices remotely triggered via image-guided ultrasound heating
Source: Nat Commun. 2024 Feb 6;15:1123. doi: 10.1038/s41467-024-45437-2 (PMC10847440; doi:10.1038/s41467-024-45437-2)
Supplement: Supplementary file 1 — Supplementary Information [file 41467_2024_45437_MOESM1_ESM.pdf]

Supporting Information

Remotely triggered shape recovery of shape memory device in large animal models via image-guided ultrasound heating

Yang Zhu[#,*], Kaicheng Deng[#], Jianwei Zhou[#], Chong Lai[#], Zuwei Ma, Hua Zhang, Jiazhen Pan, Liyin Shen, Matthew Bucknor, Eugene Ozhinsky, Seungil Kim, Guangjie Chen, Sang-ho Ye, Yue Zhang, Donghong Liu, Changyou Gao, Yonghua Xu[*], Huanan Wang[*], William R. Wagner[*]

[*] Yang Zhu, Huanan Wang, Yonghua Xu, William R. Wagner

Email: zhuyang@zju.edu.cn, hnwang@zju.edu.cn, howardyonghua@yeah.net, wagnerwr@upmc.edu

This PDF file includes:

Supplementary text

Figures S1 to S7

Tables S1 to S4

Other supplementary materials for this manuscript include the following:

Movies S1

Movies S2

Movies S3

Movies S4

Supporting Information Text

Shape recovery of PUU-PCL devices

Fe_3O_4 nanoparticles (30 wt% of PUU-PCL) were added to the PUU solution. The mixture was cast and dried on a metal wire to obtain stents with about 2.5 mm diameter and 18 cm length. The stents were heated to coil one end and increase the stent diameter of stent by balloon expansion, followed by cooling to room temperature for immobilization. Then the J-shaped coil was straightened, and the stent shrank to its original diameter when triggered at high temperature, meanwhile the process was recorded.

The above mixture was cast and dried on a metal wire to obtain 2.5 mm diameter Fe_3O_4 nanoparticle incorporated PUU-PCL tubes. The ends of tube were heated to fold, followed by cooling to room temperature for immobilization. In this way, PUU-PCL tube without dye solution was obtained. The shape recovery of this tube was triggered by 808 nm laser with output power of 1 W (MDL-N-808-8W, Changchun New Industries Optoelectronics Tech, China) in vitro to mimic HIFU remote heating, recording by camera.

Preparation of Fe_3O_4 nanoparticle incorporated PUU-PCL tube with dye solution was very similar with above step. One end of PUU-PCL tube was folded and immobilized, the dye solution was immediately loaded into the tube, followed by folding and immobilization of the other end. The shape recovery of this tube was triggered in warm water at 55 ^{o}C in vitro, recorded by camera. In animal experiments, the above tubes were assembled onto the PDMS patch. Shape recovery in sheep study was described below in the animal experiment section.

Cytocompatibility of PUU-PCL

Smooth muscle cells (A7r5, Cell Bank of Typical Culture Collection of Chinese Academy of Sciences) were used to evaluate the biocompatibility of PUU-PC in vitro and in vivo. We cultured smooth muscle cells on the surface of PUU-PCL films. After 1 or 7 days of culture on the films, live/dead staining (Calcein-AM and propidium iodid, Beyotime Biotechnology, China) and MTS assay (Cell counting kit-8, Beyotime Biotechnology, China) were employed.

Degradation of PUU-PCL

PUU-PCL was dissolved in hexafluoroisopropanol, the solution was cast and dried on a Teflon mold to obtain PUU-PCL films, which were cut into small films with the same size. The films were placed in 37 ºC PBS (n = 5), and the weight loss of PUU-PCL films were measured at scheduled time points.

Biocompatibility of PUU-PCL

PUU-PCL and Fe_3O_4 incorporated PUU-PCL was processed into tubes of the same size as commercially available stent by dip-coating. PUU-PCL tubes, Fe_3O_4 incorporated PUU-PCL tubes and commercially available stents were subcutaneously implanted in rats for 4 weeks (n = 4 per group). Four weeks later, the blood was collected for evaluation of hepatorenal safety. Then the subcutaneous tissue around the implanted tube and vital organs, including the liver and kidney, were harvested for histopathologic examination.

Acute systemic toxicity was evaluated by Hangzhou Tigermed Testing, a third-party institution possesses China National Accreditation Service (CNAS) qualification and China Metrology Accreditation (CMA) qualification, issued by CNAS/CMA certified laboratory test report (ISO/IEC 17025). The test was performed according to the ISO 10993.11-2017 standards for Biological evaluation of medical devices - Part 11: Tests for systemic toxicity. The test article was extracted in 0.9% sodium chloride (SC) and cottonseed oil (CSO), the control vehicle would be the same method as test extract but without test article. Each polar extract was intravenously injected to five ICR mice. Each non-polar extract was intraperitoneally injected to five test mice. The general state, toxicity and number of dead animals were observed at 4h, 24h, 48h and 72h after injection, and the animal weight was recorded daily. Clinic pathological and gross pathology evaluations were performed. The evaluated clinical symptoms were listed in **Table S1**. If any gross pathology abnormality is observed at autopsy, histopathological examination is made. Body weight of all the experimental animals increased obviously, and there was no obvious abnormality in diet and growth state. Clinical observations and body weights data are shown in **Table S2**. Clinical pathology and gross pathology evaluation: Not performed as there were no dead animals and no clinical symptoms during the experiment.

USgHIFU triggered shape recovery of moving devices and dye release on sheep liver

This animal experiment was approved by the Guidelines of Animal Care and Use Committees of Zhejiang University (ZJU20230348). In the animal experiment of HIFU triggered shape recovery of a PUU-PCL device on sheep liver (Hu sheep, female, 1 year old, weighed 40.0 kg). Ceftiofur solution was used for anti-infection. Lactated Ringer's solution was used to replenish body fluids. The above patch integrated with PUU-PCL tubes was fixed on the liver of a sheep one day before HIFU operation, monitoring sheep physical condition and indicators. The surgery was performance next day. The anesthetized sheep was placed on the operation table in a lateral position and the body was adjusted for ultrasonic probe attachment. The focus point of HIFU was adjusted onto the Fe_3O_4 nanoparticle incorporated PUU-PCL tubes. The Fe_3O_4 nanoparticle incorporated PUU-PCL tubes were heated under line scan mode at a power of 400 W for 10 s from the distal end of the device to the proximal end. After HIFU heating, the patch was taken out from sheep liver for observation. Sheep was ventilated for an extra hour before they resumed spontaneous breathing. The HIFU parameters used in the animal studies are listed in **Table S4**.

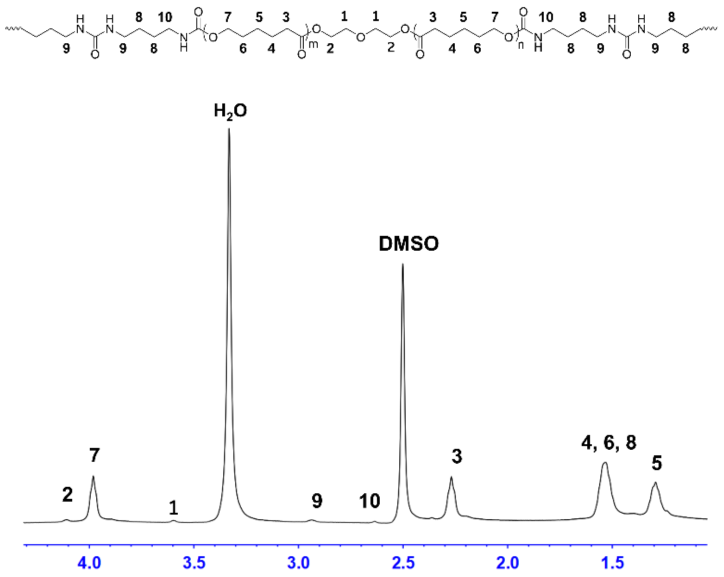

Figure S1. ^{1}H NMR spectrum of PUU-PCL.

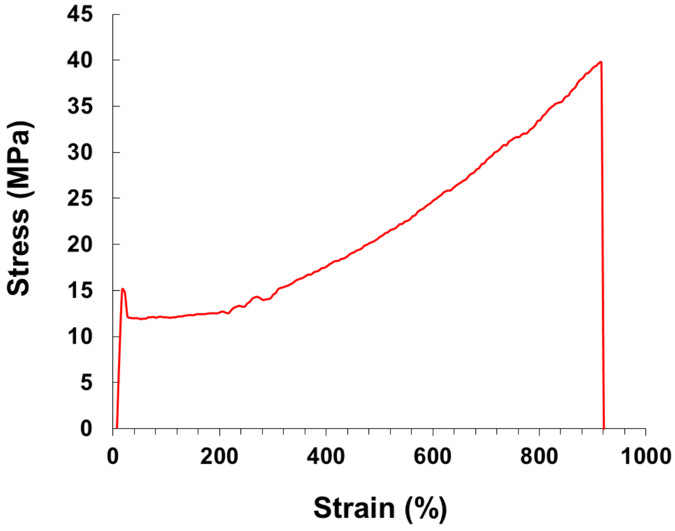

Figure S2. Stress-strain curve of PUU-PCL in uniaxial stretch test.

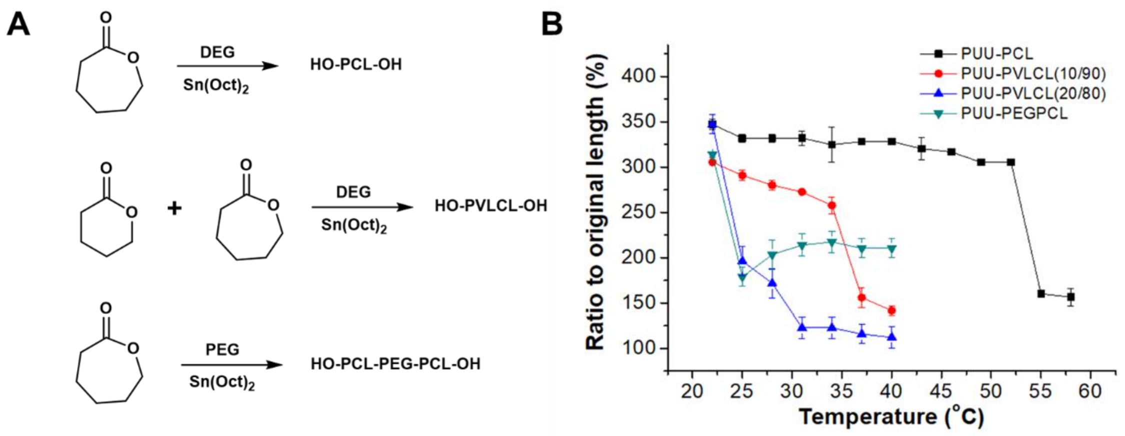

Figure S3. Synthesis and transition temperature of different PUUs. (A) Synthesis of different soft segments of PUUs, including PCL diol, PVLCL diol and PCL-PEG-PCL diol. (B) Transition temperature of PUUs with different soft segments. Copolymerization with VL, PEG lowered the transition temperature to below body temperature, (n = 3).

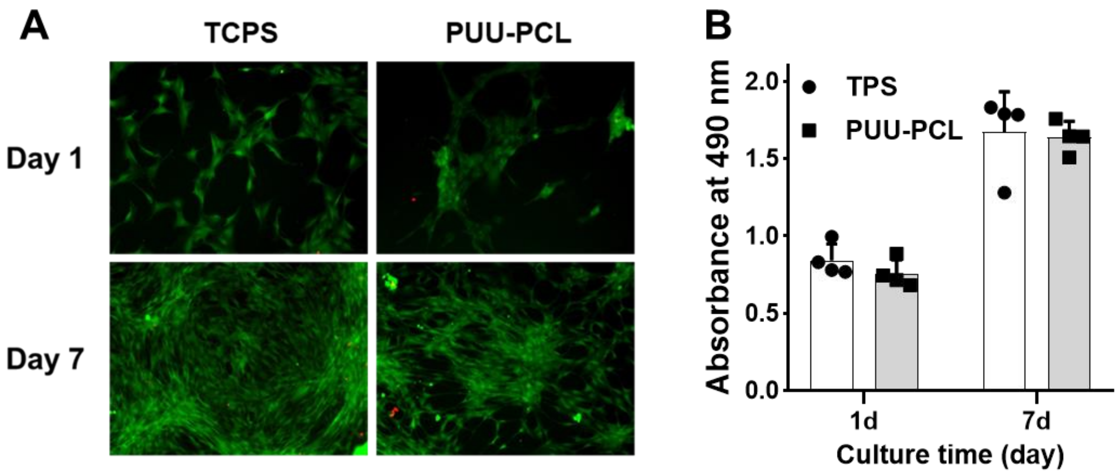

Figure S4. Cytocompatibility of PUU-PCL. (A) Live/dead staining. (B) MTS assay (n = 4).

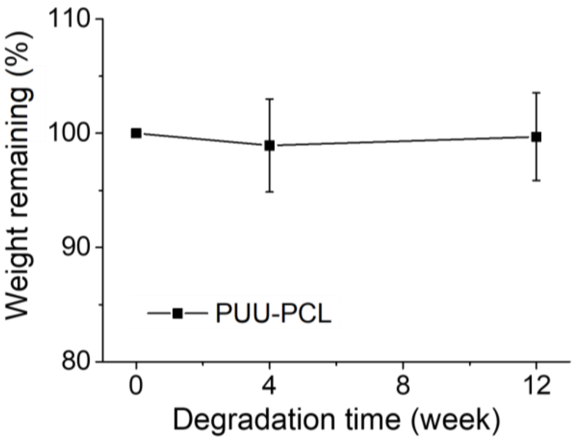

Figure S5. Degradation of PUU-PCL films in PBS (n = 5).

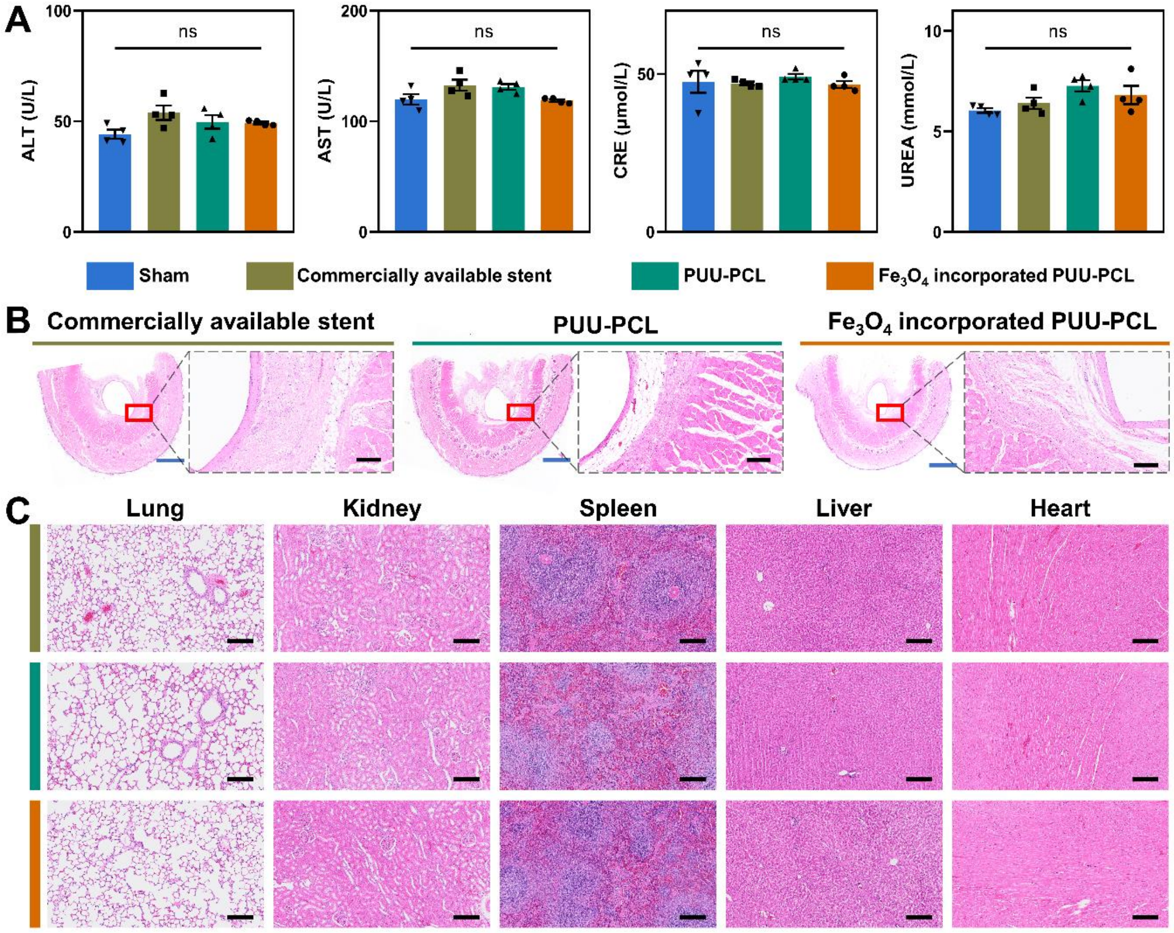

Figure S6. (A) Hepatorenal safety of implanted tubes by blood detection (n = 4). Statistical significance was calculated using one-way ANOVA with Tukey's test, and data are presented as means ± SEM. ns ＞ 0.05. (B) Histopathologic examination of subcutaneous tissue around the implanted tube with larger magnification insets (representative for n = 4 biologically independent samples). Scale bar (blue) = 2 mm. Scale bar (black) = 200 μm. (C) Histopathologic examination of vital organs (representative for n = 4 biologically independent samples). Scale bar = 200 μm.

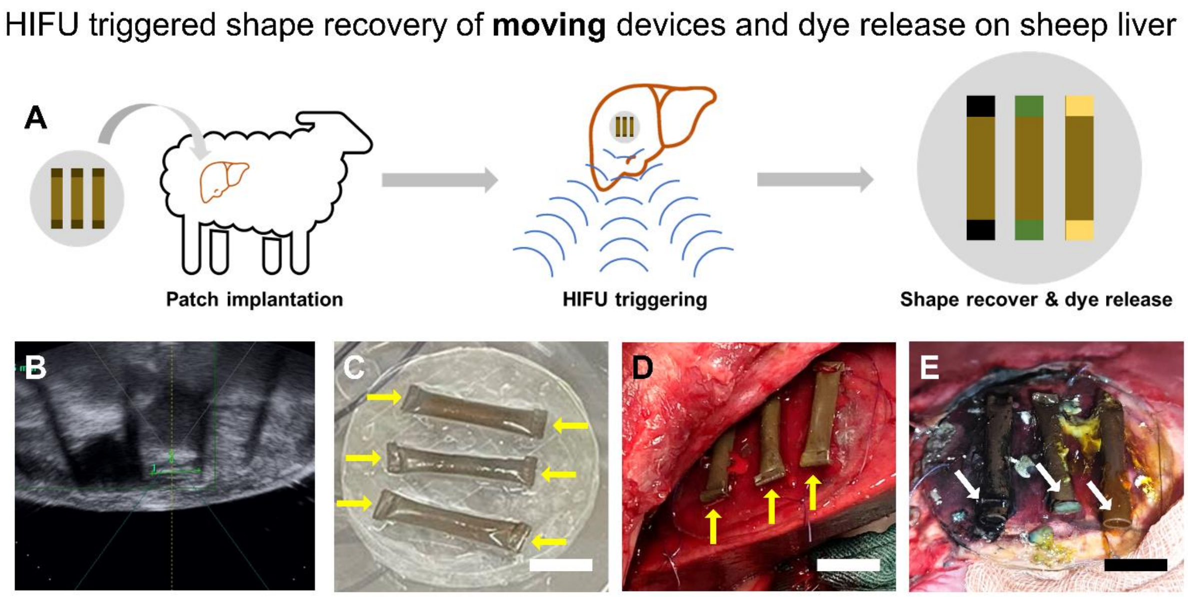

Figure S7. USgHIFU triggered in vivo shape recovery of deeply implanted moving devices and dye solution release. (A) Scheme of animal experiment including patch implantation, HIFU triggered shape recovery, and dye release. (B) B mode ultrasound image of PUU-PCL tubes (loaded with Fe_3O_4 nanoparticles, fixed on a PDMS patch) on moving sheep liver. The plane of PUU-PCL tubes is indicated by the green line. (C) Sealed PUU-PCL tubes loaded with dye solution (black, green, and yellow dye in different tubes). Yellow arrows: tube ends sealed at room temperature. (D) Dye solution loaded tubes implanted on a sheep liver. The tubes moved with the liver at the frequency of sheep respiration. (E) USgHIFU treated tubes. The sealed ends reopened, as pointed by the white arrows. All three dyes released from the tubes can be observed. Scale bar = 1 cm.

Table S1. Observation of toxic reactions

Clinical observation	Observed sign	Involved system(s)
Respiratory	Dyspnea (abdominal breathing, gasping), apnoea, cyanosis, tachypnea, nostril discharges	CNS, pulmonary, cardiac
Motor activities	Decrease/increase somnolence, loss of righting, catalepsy, ataxia, unusual locomotion, prostration, tremors, fasciculation	CNS, somatomotor, sensory, neuromuscular, autonomic
Convulsion	Clonic, tonic, tonic-clonic, asphyxial, opisthotonos	CNS, neuromuscular, autonomic, respiratory
Reflexes	Corneal, righting, myotact, light, startle reflex	CNS, sensory, autonomic, neuromuscular,
Ocular signs	Lacrimation, miosis, mydriasis, exophthalmos, ptosis, opacity, iritis, conjunctivitis, chromodacryorrhea, relaxation of nictitating membrane	Autonomic, irritation
Cardiovascular signs	Bradycardia, tachycardia, arrhythmia, vasodilation, vasoconstriction,	CNS, autonomic, cardiac, pulmonary
Salivation	Excessive	Autonomic
Piloerection	Rough hair	Autonomic
Analgesia	Decrease reaction	CNS, sensory
Muscle tone	Hypotonia, hypertonia	Autonomic
Gastrointestinal	Soft stool, diarrhoea, emesis, diuresis, rhinorrhea	CNS, autonomic, sensory, GI motility, kidney
Skin	Oedema, erythema	Tissue damage, irritation

Table S2. Body weight at different times and clinical observation results of test animals after treatment

Group	Treatment pathway	Animals number	Sex	Weight(g)				Clinical observation				
				Initial	24h	48h	72h	Immediate	4h	24h	48h	72h
Test group	IV.	231083	Female	18.5	19.4	20.4	21.2	—	—	—	—	—
		231097		19.2	19.8	21.0	22.0	—	—	—	—	—
		231073		19.6	21.3	23.0	24.4	—	—	—	—	—
		231096		18.7	19.5	20.5	21.4	—	—	—	—	—
		231100		18.2	18.6	19.6	20.9	—	—	—	—	—
	IP.	231072	Female	18.7	19.8	20.2	21.1	—	—	—	—	—
		231082		18.3	19.3	20.0	20.7	—	—	—	—	—
		231079		19.9	20.2	20.9	22.0	—	—	—	—	—
		231102		19.0	19.7	20.7	21.6	—	—	—	—	—
		231088		20.8	21.1	22.3	23.2	—	—	—	—	—
Control group	IV.	231084	Female	18.3	19.6	20.4	21.9	—	—	—	—	—
		231074		18.7	19.4	20.4	21.7	—	—	—	—	—
		231103		20.4	21.6	22.6	24.2	—	—	—	—	—
		231086		20.9	21.8	22.7	23.9	—	—	—	—	—
		231104		19.9	21.3	22.1	23.5	—	—	—	—	—
	IP.	231071	Female	19.3	20.1	20.7	22.1	—	—	—	—	—
		231095		20.6	21.5	22.7	23.6	—	—	—	—	—
		231085		18.4	20.0	20.8	21.5	—	—	—	—	—
		231093		19.8	20.4	21.4	22.3	—	—	—	—	—
		231105		21.8	23.1	24.3	25.1	—	—	—	—	—

IV. intravenous injection. IP. intraperitoneal injection. “—”: the animal is normal after injection.

Table S3. Blood and urine test results before and after HIFU procedure

	Before surgery	1 day after surgery	2 days after surgery	Normal range
WBC	12.02	18.85	16.64	5.05-16.76 K/μL
NEU	8.90	12.01	10.18	2.95-11.64 K/μL
ALP	66	111	113	20-150 U/L
ALT	32	57	43	10-118 U/L
BUN	15	11	4	7-25 mg/dL
CRE	0.7	0.6	0.5	0.3-1.4 mg/dL
BLD	Negative	250 Ery/μL	250 Ery/μL	Negative
LEU	Negative	500 Leu/μL	500 Leu/μL	Negative
Color of urine	Transparent yellow	Transparent yellow	Dark yellow	No red color

Table S4. HIFU parameters in the animal studies

Model	Mode	Power	Energy	Pulse length	Duration of prescription	Duty cycle
Canine, bladder	line scan	420 W	3360 J	20 mm	8 s	100%
Sheep, liver	line scan	400 W	4000 J/scan	20 mm	10 s/scan	100%
