# Supplementary material for: Shape-recovery of implanted shape-memory devices remotely triggered via image-guided ultrasound heating
Source: Nat Commun. 2024 Feb 6;15:1123. doi: 10.1038/s41467-024-45437-2 (PMC10847440; doi:10.1038/s41467-024-45437-2)
Supplement: Supplementary file 3 — Description of Additional Supplementary Files [file 41467_2024_45437_MOESM3_ESM.pdf]

<u>Description of Additional Supplementary Files</u>

Supplementary Movie Legends

Supplementary Movie 1: Shape recovery of single-J ureteral stent of Fe3O4 nanoparticles incorporated PUU-PCL.

Supplementary Movie 2: Temperature triggered device shape recovery and dye release in vitro.

Supplementary Movie 3: Laser triggered shape recovery in vitro.

Supplementary Movie 4: In vivo shape recovery of flag shaped device of Fe3O4 nanoparticles incorporated PUU-PCL
