# Supplementary material for: Shape-recovery of implanted shape-memory devices remotely triggered via image-guided ultrasound heating
Source: Nat Commun. 2024 Feb 6;15:1123. doi: 10.1038/s41467-024-45437-2 (PMC10847440; doi:10.1038/s41467-024-45437-2)
Supplement: Supplementary file 8 — Reporting Summary [file 41467_2024_45437_MOESM8_ESM.pdf]

nature portfolio

Corresponding author(s): Yang Zhu

Last updated by author(s): Dec 11, 2023

# Reporting Summary

Nature Portfolio wishes to improve the reproducibility of the work that we publish. This form provides structure for consistency and transparency in reporting. For further information on Nature Portfolio policies, see our Editorial Policies and the Editorial Policy Checklist.

## Statistics

For all statistical analyses, confirm that the following items are present in the figure legend, table legend, main text, or Methods section.

| n/a | Confirmed | |
|---|---|---|
| ☐ | ☒ | The exact sample size (*n*) for each experimental group/condition, given as a discrete number and unit of measurement |
| ☐ | ☒ | A statement on whether measurements were taken from distinct samples or whether the same sample was measured repeatedly |
| ☐ | ☒ | The statistical test(s) used AND whether they are one- or two-sided<br>*Only common tests should be described solely by name; describe more complex techniques in the Methods section.* |
| ☒ | ☐ | A description of all covariates tested |
| ☐ | ☒ | A description of any assumptions or corrections, such as tests of normality and adjustment for multiple comparisons |
| ☐ | ☒ | A full description of the statistical parameters including central tendency (e.g. means) or other basic estimates (e.g. regression coefficient) AND variation (e.g. standard deviation) or associated estimates of uncertainty (e.g. confidence intervals) |
| ☐ | ☒ | For null hypothesis testing, the test statistic (e.g. *F*, *t*, *r*) with confidence intervals, effect sizes, degrees of freedom and *P* value noted<br>*Give P values as exact values whenever suitable.* |
| ☒ | ☐ | For Bayesian analysis, information on the choice of priors and Markov chain Monte Carlo settings |
| ☒ | ☐ | For hierarchical and complex designs, identification of the appropriate level for tests and full reporting of outcomes |
| ☒ | ☐ | Estimates of effect sizes (e.g. Cohen's *d*, Pearson's *r*), indicating how they were calculated |

*Our web collection on statistics for biologists contains articles on many of the points above.*

## Software and code

Policy information about availability of computer code

| | |
|---|---|
| Data collection | 300 MHz spectrometer(Bruker,German), DP2-BSW software (Olympus, VS200, USA), DSC-60 instrument (Shimadzu, Japan), MicroForce Testing Workstation (MTS Tytron 250, USA), A Magnetic resonance guided high intensity focused ultrasound (MRgHIFU) system (ExAblate2000, Insightec, Haifa, Israel), Type JC USgHIFU (Haifu, China). |
| Data analysis | Image-Pro Plus 6.0, GraphPad Prism 8.0.1, Origin 2018, |

For manuscripts utilizing custom algorithms or software that are central to the research but not yet described in published literature, software must be made available to editors and reviewers. We strongly encourage code deposition in a community repository (e.g. GitHub). See the Nature Portfolio guidelines for submitting code & software for further information.

## Data

Policy information about availability of data

All manuscripts must include a data availability statement. This statement should provide the following information, where applicable:

- Accession codes, unique identifiers, or web links for publicly available datasets
- A description of any restrictions on data availability
- For clinical datasets or third party data, please ensure that the statement adheres to our policy

All relevant data related to this manuscript are available on request from the authors on reasonable request.

## Research involving human participants, their data, or biological material

Policy information about studies with human participants or human data. See also policy information about sex, gender (identity/presentation), and sexual orientation and race, ethnicity and racism.

| | |
|---|---|
| Reporting on sex and gender | No human research participants was involved in the study. |
| Reporting on race, ethnicity, or other socially relevant groupings | No human research participants was involved in the study. |
| Population characteristics | No human research participants was involved in the study. |
| Recruitment | No human research participants was involved in the study. |
| Ethics oversight | No human research participants was involved in the study. |

Note that full information on the approval of the study protocol must also be provided in the manuscript.

# Field-specific reporting

Please select the one below that is the best fit for your research. If you are not sure, read the appropriate sections before making your selection.

☒ Life sciences ☐ Behavioural & social sciences ☐ Ecological, evolutionary & environmental sciences

For a reference copy of the document with all sections, see nature.com/documents/nr-reporting-summary-flat.pdf

# Life sciences study design

All studies must disclose on these points even when the disclosure is negative.

| | |
|---|---|
| Sample size | Large animal experiments were performed to verify the feasibility of HIFU-triggered shape recovery of the shape memory device in large animal model, so only one animal was used in large animal experiments. |
| Data exclusions | No data were excluded from the analyses. |
| Replication | Large animal experiments were performed to verify the feasibility of HIFU-triggered shape recovery of the shape memory device in large animal model, so only one animal was used in large animal experiments. |
| Randomization | The sample in animal experiment was randomized. |
| Blinding | All data collection and analysis were blinded to group allocation |

# Reporting for specific materials, systems and methods

We require information from authors about some types of materials, experimental systems and methods used in many studies. Here, indicate whether each material, system or method listed is relevant to your study. If you are not sure if a list item applies to your research, read the appropriate section before selecting a response.

### Materials & experimental systems

| n/a | Involved in the study | |
|---|---|---|
| ☒ | ☐ | Antibodies |
| ☒ | ☐ | Eukaryotic cell lines |
| ☒ | ☐ | Palaeontology and archaeology |
| ☐ | ☒ | Animals and other organisms |
| ☒ | ☐ | Clinical data |
| ☒ | ☐ | Dual use research of concern |
| ☒ | ☐ | Plants |

### Methods

| n/a | Involved in the study | |
|---|---|---|
| ☒ | ☐ | ChIP-seq |
| ☒ | ☐ | Flow cytometry |
| ☒ | ☐ | MRI-based neuroimaging |

## Animals and other research organisms

Policy information about studies involving animals; ARRIVE guidelines recommended for reporting animal research, and Sex and Gender in Research

| | |
|---|---|
| Laboratory animals | ICR mice (female, 5 weeks old, weighed 18-22 g)Beagle (female, 2 years old, weighed 13.0 kg) and Hu Sheep (female, 1 year old, weighed 40.0 kg) were used as animal models in this work. The ICR mice were kept at a temperature of 20-24 degrees Celsius, a humidity of 40-70%, and an alternating time of 12/12 between light and dark. |
| Wild animals | No wild animals were used in the study |
| Reporting on sex | Sex was not considered in the study. |
| Field-collected samples | No field collected samples were used in the study |
| Ethics oversight | The Institutional Animal Care and Use Committee of Zhejiang University. |

Note that full information on the approval of the study protocol must also be provided in the manuscript.

## Plants

| | |
|---|---|
| Seed stocks | No plants were involved in the study. |
| Novel plant genotypes | No plants were involved in the study. |
| Authentication | No plants were involved in the study. |
